# Orbital and adnexal tuberculosis: a case series from a South Indian population

**DOI:** 10.1186/1869-5760-4-12

**Published:** 2014-05-22

**Authors:** Kalpana Babu, Moupia Mukhopadhyay, Soumya S Bhat, JT Chinmayee

**Affiliations:** 1 Vittala International Institute of Ophthalmology, Bangalore 560085, India; 2 Prabha Eye Clinic and Research Centre, 504, 40th Cross, Jayanagar 8th Block, Bangalore 560070, India

**Keywords:** Orbital tuberculosis, *Mycobacterium tuberculosis*, Proptosis, Antitubercular therapy

## Abstract

**Background:**

Orbital tuberculosis (OTb) is rare and may be regarded as a manifestation of extrapulmonary tuberculosis. We report an interesting case series of six patients with varied presentations of orbital and adnexal tuberculosis in a South Indian patient population.

**Results:**

A retrospective, interventional case series of six patients diagnosed with orbital and adnexal tuberculosis on the basis of clinical, radiological and histopathological evaluations between 2010 and 2013 was performed. Among the six patients with histopathologically proven OTb, five were women. The varied presentations included tubercular dacryoadenitis (two cases), classical periostitis (two cases), OTb with bone involvement (one case) and ocular adnexal tuberculosis (one case). Systemic involvement was seen in one case. All cases were treated with a regimen of antitubercular therapy (ATT).

**Conclusions:**

OTb, though rare, should form a part of the differential diagnosis of orbital lesions in a high tuberculosis (TB) endemic country like ours. Biopsy still remains the mainstay of diagnosis.

## Background

Recent years have demonstrated an increasing trend in the diagnosis of ocular tuberculosis, due to the increased awareness of the disease and availability of better diagnostic modalities. In contrast, orbital tuberculosis (OTb) is rare but still forms an important differential diagnosis of orbital mass lesions in a high tuberculosis (TB) endemic setting like ours.

OTb represents an extrapulmonary form of tuberculosis and may arise either by haematogenous route or spread directly from the paranasal sinuses. Diagnosis is usually by biopsy and supported by ancillary investigations like the Mantoux test, radiology and molecular diagnostic techniques like the polymerase chain reaction (PCR). In this case series, we look at the clinical profile of six patients diagnosed with orbital and adnexal tuberculosis.

## Methods

Retrospective chart review of six patients with histopathologically proven OTb between 2010 and 2013 was performed.

## Results

Five out of the six cases in our series were women and in the middle age group (range, 15 to 56 years; mean, 38 years).

### Case 1

**Figure 1:**
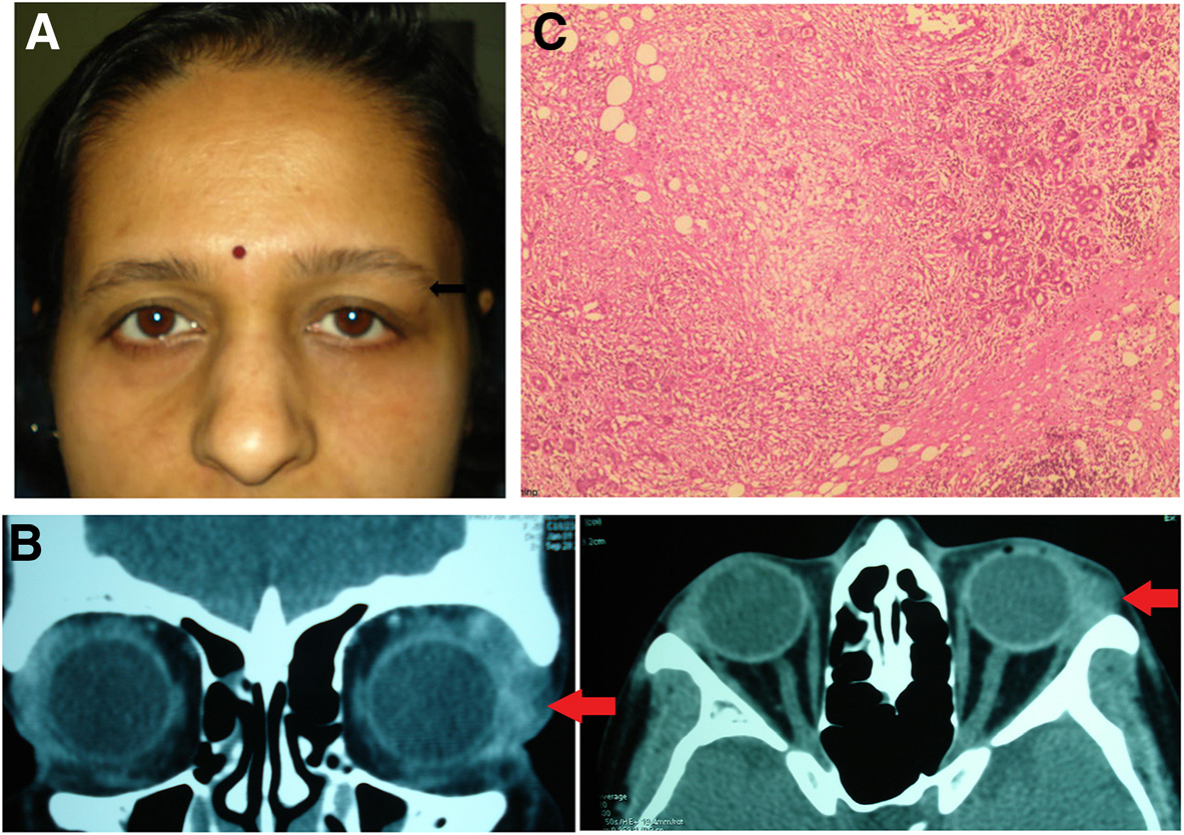
**Examination results of case 1. (A)** Face photograph of case 1 showing fullness along the lateral orbital rim with ptosis especially laterally (arrow). **(B)** External photograph showing sites of abscess drainage (scars) in the supraclavicular region and arm (arrows). **(C)** Computed tomography scan of the orbits showing lytic lesion in the bone with sequestration. **(D)** Photograph showing the pus collected and the chronic granulomatous inflammation on histopathology showing caseation necrosis.

A 15-year-old girl presented with a history of painless, progressive swelling on the left side of the face of 2 months duration. She had a history of drainage of abscesses in the neck and arm, following which she was on antitubercular therapy for 3 months, which she eventually discontinued abruptly. On examination, fullness along the left lateral orbital rim with ptosis (especially laterally) and limitation of abduction in the left eye were noted (Figure 1A). Her BCVA was 6/6 in both eyes. Slit lamp, fundus and intraocular pressure evaluations were normal in both eyes. Systemic examination revealed scars in the supraclavicular region and arm (Figure 1B). Computed tomography (CT) of the orbits revealed a well-defined lytic lesion along the lateral orbital wall with central sequestrum and associated peripherally enhancing abscess with minimal periosteal reaction suggestive of osteomyelitis (Figure 1C). Laboratory investigations revealed an increased erythrocyte sedimentation rate (ESR) of 60 mm/h, a positive Mantoux test and a normal chest X-ray. The abscess was drained, and the wall of the abscess cavity was sent for microbiological and histopathological examination. Chronic granulomatous inflammation with caseation necrosis was seen on histopathology (Figure 1D). Although the Gomori methenamine silver (GMS) staining, acid fast bacilli (AFB) staining and culture for *Mycobacterium tuberculosis* (MTb) were negative, PCR was positive for MTb. She was started on a second-line antitubercular therapy (ATT) which included daily intramuscular injections of streptomycin 0.75 mg for 6 months, ethionamide tablet 250 mg twice daily, cycloserine tablet 250 mg twice daily and ofloxacin tablet 400 mg once daily for 1.5 years. At the last follow-up, the lesion at the lateral orbital rim had resolved completely.

### Case 2

**Figure 2:**
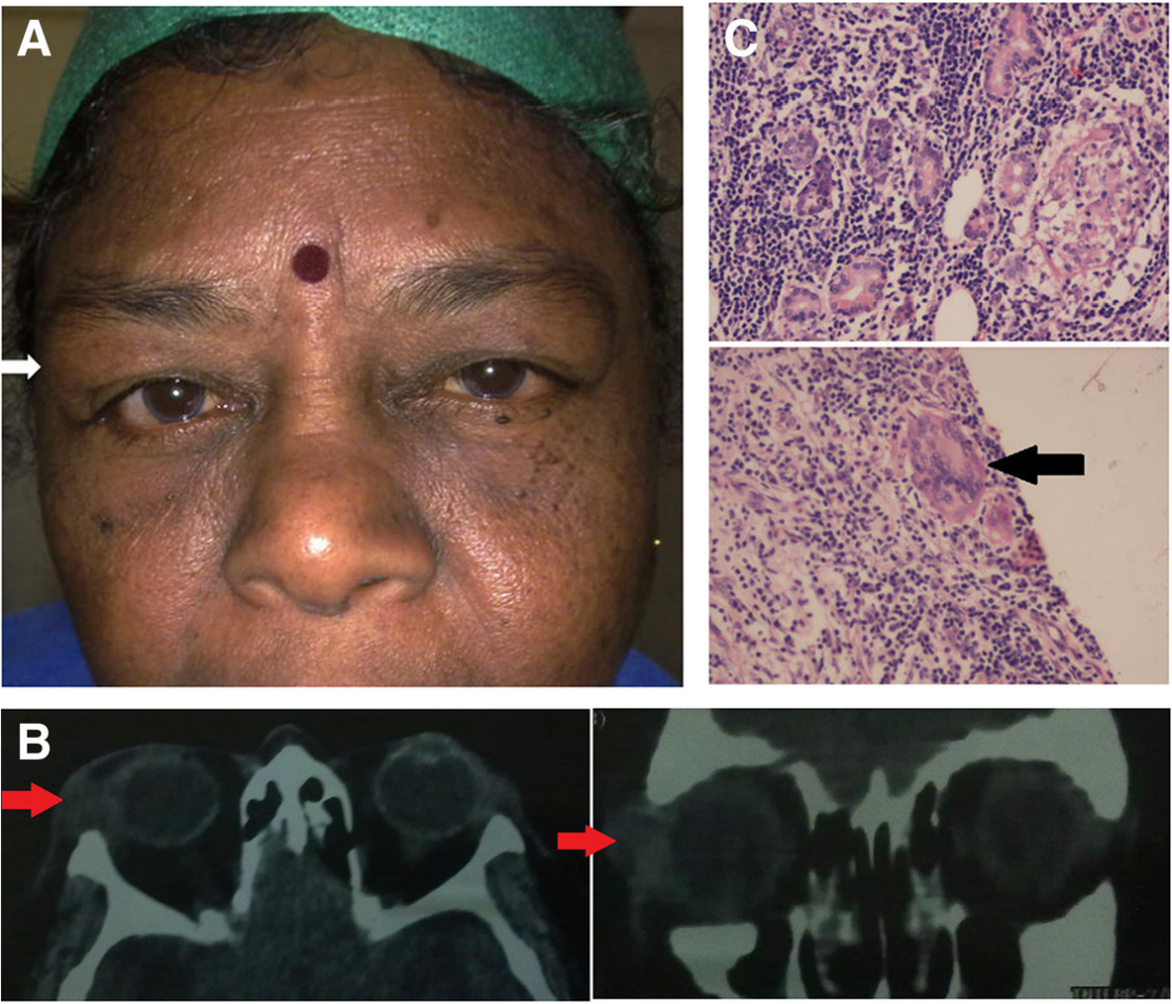
**Examination results of case 2. (A)** Face photograph of case 2 showing fullness below the lateral part of the left eye brow in the lacrimal gland area (arrow). **(B)** CT scan showing diffuse enlargement of the lacrimal gland, without bony erosion or bony remodelling. **(C)** Photomicrograph showing granulomatous inflammation with caseation necrosis (arrow).

A 40-year-old lady presented with a swelling below the left eyebrow of 1 month duration. Examination revealed a firm, palpable mass in the region of the lacrimal gland (Figure 2A). Ocular evaluation was normal in both eyes. CT scan showed diffuse enlargement of the lacrimal gland with no bony remodelling or moulding around the globe (Figure 2B). The excised mass showed granulomatous inflammation with caseation necrosis on histopathology (Figure 2C). GMS, Ziehl-Neelson (ZN) staining and PCR for MTb were negative. Laboratory evaluation showed an ESR of 14 mm/h, normal chest radiography including CT thorax and a positive Mantoux test. She was started on four-drug regimen of ATT (isoniazid, rifampicin, ethambutol and pyrazinamide). At the end of 1 year, she is doing well with no recurrence of inflammation.

### Case 3

**Figure 3:**
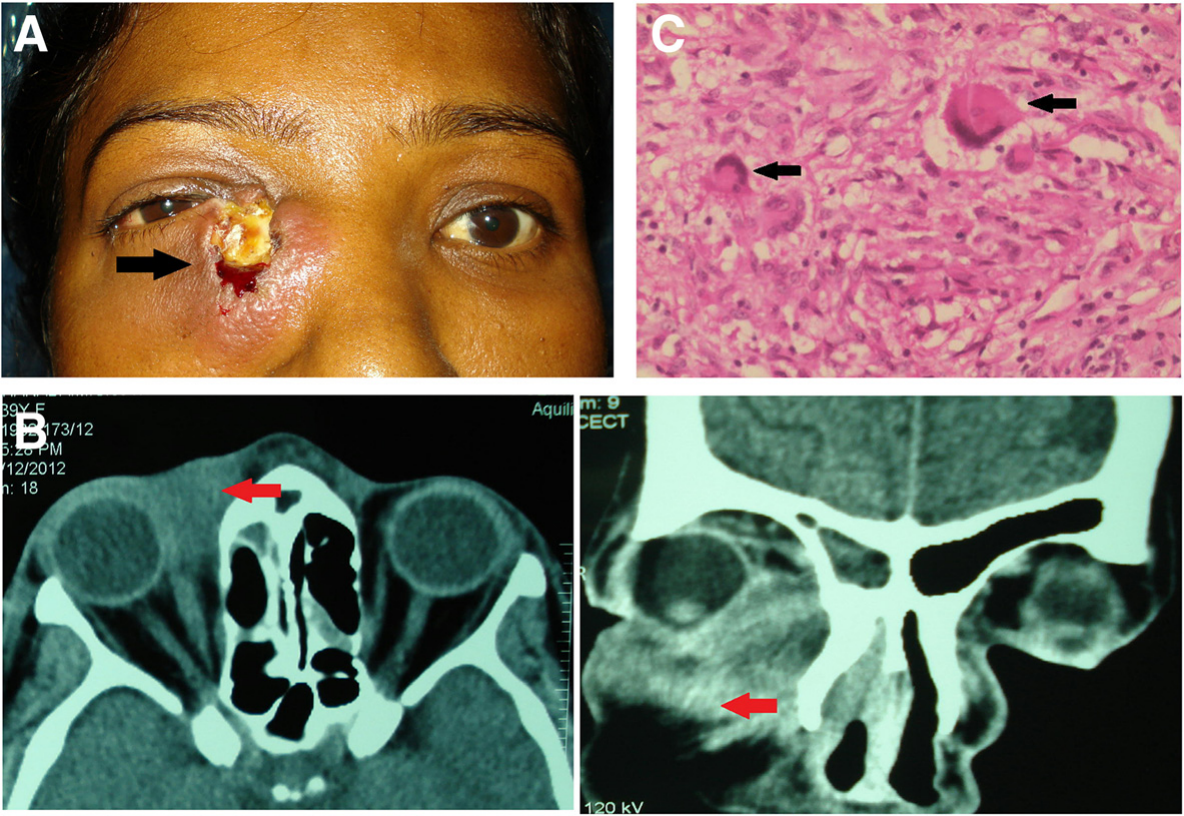
**Examination results of case 3. (A)** Face photograph of case 3 showing fullness below the lateral part of the right eye brow. **(B)** CT scan showing diffuse enlargement of the lacrimal gland (arrows) without any bony erosion or remodelling. **(C)** Microphotograph showing the chronic granulomatous inflammation with giant cells (arrow) and caseation necrosis (H&E).

A 56-year-old lady presented with swelling below the right eyebrow of 3 months duration. Examination revealed a diffuse, non-tender, firm mass in the lacrimal gland area (Figure 3A). CT scan showed diffuse enlargement of the lacrimal gland with no bony erosion or remodelling (Figure 3B). Excision biopsy showed granulomatous inflammation with caseation necrosis on histopathology (Figure 3C). GMS, ZN staining and PCR for MTb were negative. Laboratory evaluation showed an ESR of 20 mm/h, normal CT thorax and a positive Mantoux test. She was started on four-drug regimen of ATT (isoniazid, rifampicin, ethambutol and pyrazinamide). At the end of 1.5 years, she was doing well with no recurrence of mass.

### Case 4

**Figure 4:**
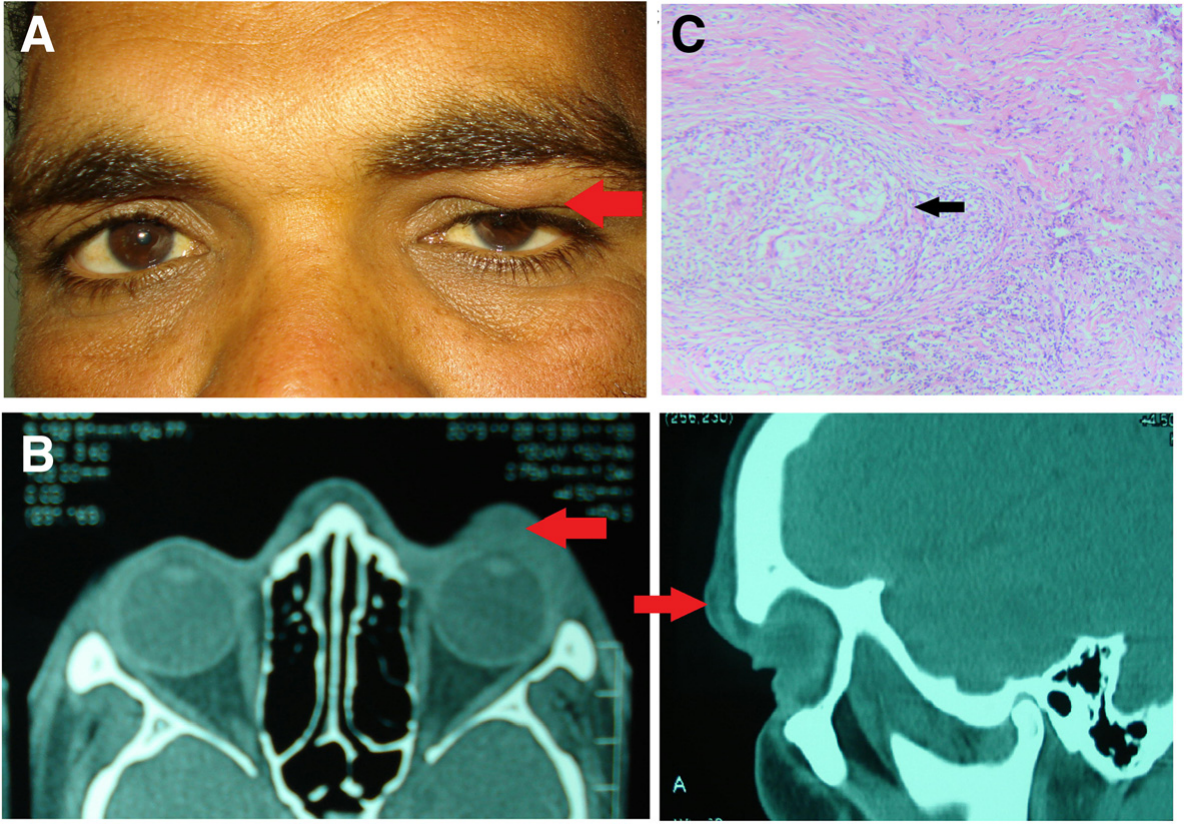
**Examination results of case 4. (A)** Face photograph of case 4 showing an indurated mass lesion with an overlying non-healing ulcer with discharge in the lacrimal sac area of the right eye (arrow). **(B)** CT scan showing soft tissue lesion in the anterior part of right medial orbit (arrow). **(C)** Photomicrograph showing chronic granulomatous inflammation with giant cells (arrow).

A 39-year-old lady was referred to our center for an indurated mass lesion with an overlying non-healing ulcer in the medial part of the right lower lid of 3 months duration (Figure 4A). CT scan of the orbits showed a soft tissue lesion in the anterior part of right medial orbit, and the lacrimal sac could not be delineated separately from the mass (Figure 4B). Histopathological examination of the abscess wall showed granulomatous inflammation with caseation (Figure 4C). ZN stain for acid fast bacilli was positive, while GMS stain for fungi was negative. Other laboratory investigations included an increase in the ESR (40 mm/h), a positive Mantoux test and a normal chest X-ray. Four-drug regimen of ATT (isoniazid, rifampicin, ethambutol and pyrazinamide) was initiated. She was doing well at the end of 6 months with no recurrence of mass or inflammation. She was lost to follow-up thereafter.

### Case 5

**Figure 5:**
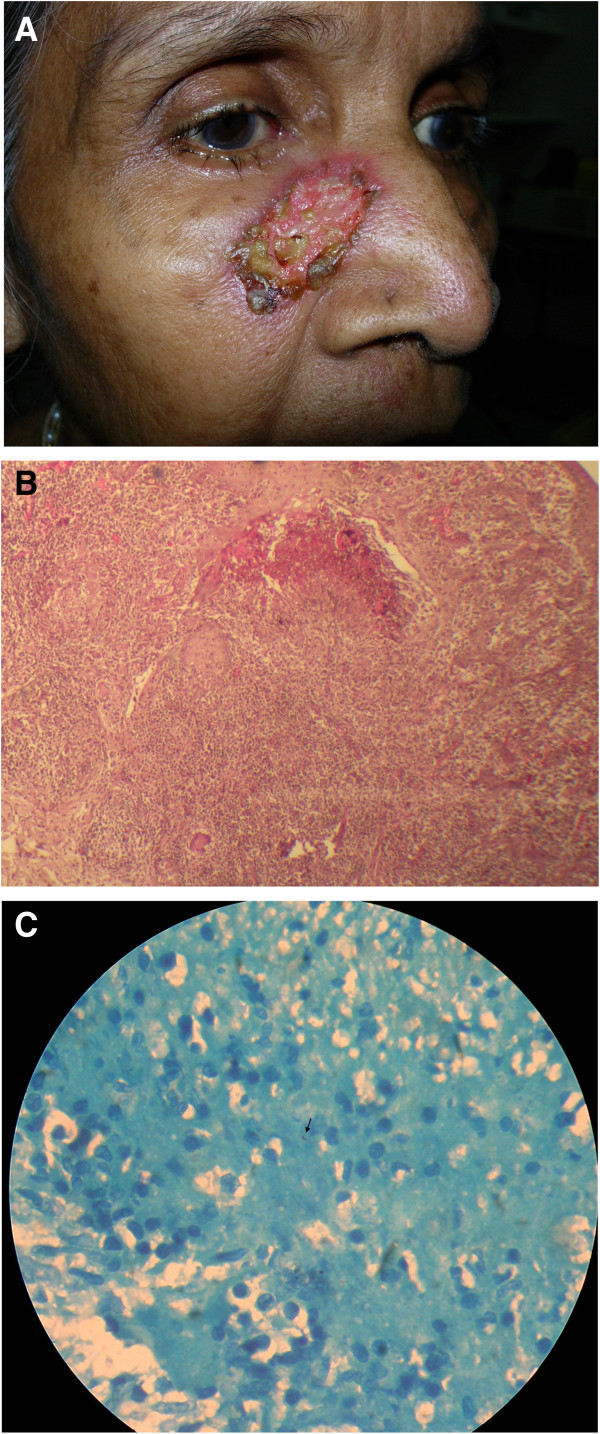
**Examination results of case 5. (A)** Face photograph of case 5 showing a preseptal swelling below the left superior orbital rim (arrow). **(B)** CT scan of the orbits showing soft tissue swelling in front of the superior orbital rim with no evidence of bony erosion (arrow). **(C)** Photomicrograph showing chronic granulomatous inflammation with giant cells and caseation necrosis (arrow).

A 28-year-old man presented with a swelling just below the left eyebrow area of 1-month duration. On examination, a firm, non-tender mass could be palpated in the inferior portion of the superior orbital rim (Figure 5A). The rest of ocular evaluation was normal. CT scan orbits showed a soft tissue swelling inseparable from the left orbital rim (Figure 5B). Excision biopsy of this lesion revealed granulomatous inflammation with caseation (Figure 5C). GMS and ZN staining were negative, while PCR for MTb was positive. Other laboratory investigations showed an increase in ESR (42 mm/h), normal CT thorax, positive Mantoux and QuantiFERON TB gold tests. He was started on four-drug regimen of ATT (isoniazid, rifampicin, ethambutol and pyrazinamide). At the last follow-up of 8 months, he is doing well with no recurrence of mass or lesion.

### Case 6

**Figure 6:**
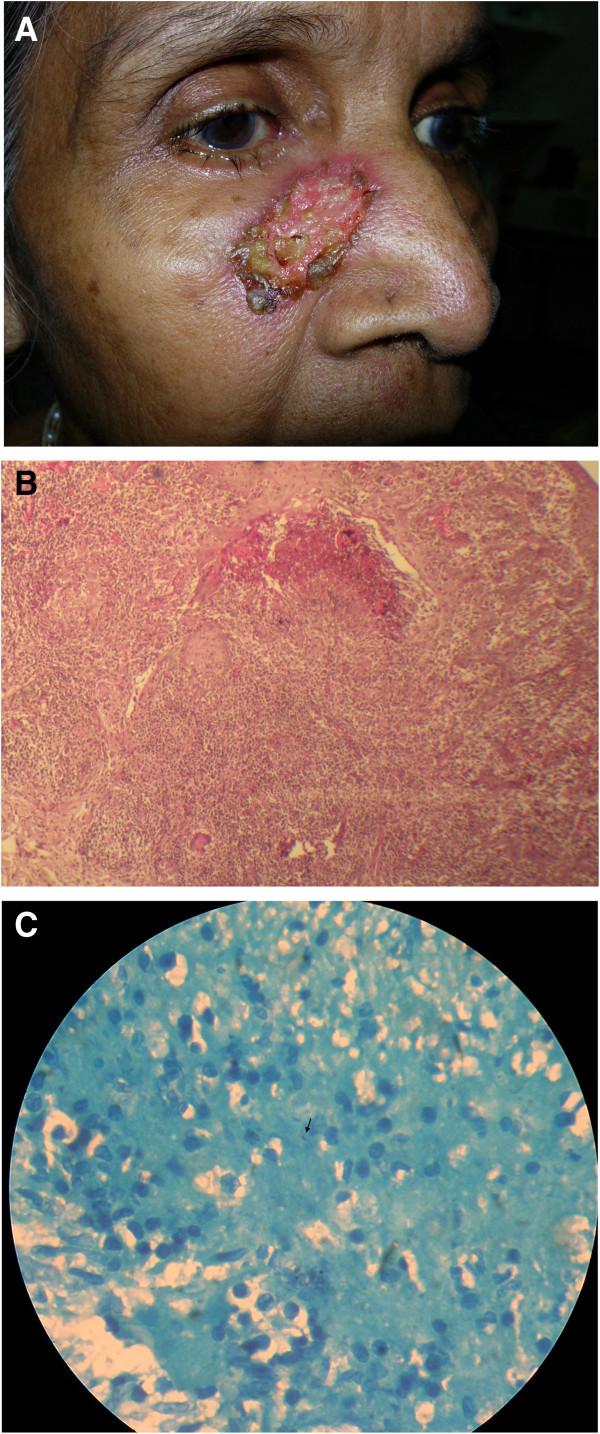
**Examination results of case 6. (A)** Face photograph of case 6 showing large ulceration on the right cheek below the right lower eyelid. Microphotograph showing **(B)** chronic granulomatous inflammation with giant cells and caseation necrosis and **(C)** positive acid fast bacilli indicated by an arrow.

A 50-year-old lady presented with a non-healing ulcer on the skin below the right lower eye lid of 4 months duration. On examination, she had a large non-healing ulcer on the right lower eyelid (Figure 6A). The ulcer was adherent to the underlying bone. CT scan of the orbits done earlier was not clear, and this lady could not afford a rescan. Histopathological examination of the excised ulcer tissue showed a granulomatous inflammation with giant cells and caseation (Figure 6B). ZN stain for AFB was positive (Figure 6C), while GMS stain for fungi was negative. There was no evidence of any malignant cells. Other laboratory evaluations showed an increase in ESR (40 mm/h), a positive Mantoux test and a normal chest X-ray. She was started on the four-drug regimen of antitubercular therapy, following which she was lost to follow-up.

Table 1 shows the salient features of the various clinical presentations in the series.

**Table 1:** Salient features of clinical presentations in our series

**Case**	**Age/sex**	**Presentation**	**Systemic disease**	**Biopsy**	**ESR**	**CXR**	**Mantoux**	**ATT**
1	15 years/F	Osteomyelitis	Supraclavicular and arm fistula scars -h/o incomplete ATT	PCR positive for MTB, AFB negative; chronic granulomatous inflammation with caseation	Increased (60 mm/h)	Normal	Positive	2nd-line ATT
2	40 years/F	Dacryoadenitis	None	Chronic granulomatous inflammation with caseation; PCR and AFB negative	Normal (14 mm/h)	Normal	Positive	1st-line ATT
3	56 years/F	Dacryoadenitis	None	Chronic granulomatous inflammation with caseation; PCR and AFB negative	Marginal increase (20 mm/h)	Normal	Positive	1st-line ATT
4	39 years/F	Periostitis	None	Chronic granulomatous inflammation with caseation; AFB positive	Increased (40 mm/h)	Normal	Positive	1st-line ATT
5	28 years/M	Periostitis	None	Chronic granulomatous inflammation with caseation; AFB negative, PCR positive for MTB	Increased (42 mm/h)	Normal	Positive	1st-line ATT
6	50 years/F	Skin ulcer adnexal tuberculosis	None	Chronic granulomatous inflammation with caseation; AFB positive	Increased (40 mm/h)	Normal	Positive	1st-line ATT

## Discussion

OTb involving the lacrimal gland was first described by Abadie in 1881 [1]. Since then, around 84 cases have been described in literature (PubMed search) and around 50 cases from India. This explains the need to consider OTb as part of differential diagnosis in orbital lesions from a Tb endemic country like ours.

Five out of the six cases in our series were women and in the middle age group (range, 15 to 56 years; mean, 38 years). This series is comparable to the existing literature on OTb.

OTb can occur as a result of haematogenous spread or contiguous spread from the neighbouring paranasal sinuses. Varied manifestations of OTb excluding oculo-adnexal tuberculosis can be grouped under five clinical groups: classical periostitis, orbital soft tissue tuberculoma or cold abscess with no bony destruction, OTb with evidence of bony involvement, orbital spread from paranasal sinuses and dacryoadenitis [1]. In our series, we found it difficult to categorise the type of OTb to any one particular group in three cases as it was difficult to figure out the initial process - whether it was a tuberculoma or a nodule followed by an ulceration or periostitis. Broadly depending on the final presentation, we could categorise them into tubercular dacryoadenitis (two cases), classical periostitis (two cases), OTb with bone involvement (one case) and ocular adnexal tuberculosis (one case). Only one of the six cases had a history of systemic tuberculosis and had received inadequate antitubercular therapy due to poor compliance. In four cases, the ESR was raised, while one case had only a marginal increase in ESR. All the cases elicited a positive Mantoux test and normal chest radiography (CT thorax was done in three cases).

The diagnosis of OTb was made by biopsy in all our cases. Although the recommended initial investigation is imaging with computed tomography, biopsy confirmation is recommended in all [2]. The characteristic biopsy finding included chronic granulomatous reaction with giant cells and caseation necrosis. Acid fast bacilli were seen in only two cases. None of the biopsy specimens grew MTb. This could be due to the paucibacillary nature of the disease. PCR on the biopsy sample was positive for MTb in only two out of the four cases in whom the PCR was done. This again could be due to the low sensitivity of the test and the paucibacilliary nature of the disease. In those cases where AFB was negative, it is important to exclude fungal infections as well.

In all our cases, the chest radiography was normal. High-resolution CT thorax probably may be more informative. Treatment requires ATT. Reports of 6 to 18 months of treatment have been described in literature [3,4]. However, in an endemic country like ours, a minimum duration of 9 to 12 months is recommended for intraocular tuberculosis [5]. The challenge lies in the compliance in taking the medications. Drug resistance is becoming a serious issue in recent years in India and is a cause of concern. Adequate counselling should form a mandatory part of the treatment.

Surgery may be diagnostic or therapeutic. If diagnosed early and treated adequately, the prognosis is good in these cases [4,6].

The number of cases of different manifestations of orbital tuberculosis in literature [1-16] are highlighted in Table 2.

**Table 2:** Types of orbital tuberculosis described in literature with the number of cases

	**Clinical groups of OTB**	**Number of cases**	**Total**
1.	Classical periostitis	24 [1], 3 [7], 1 [8]	28
2.	Orbital soft tissue tuberculoma or cold abscess, no bony destruction	18 [1], 3 [7]	21
3.	OTB with evidence of bony destruction	21 [1], 1 [6],1 [9], 2 [10]	25
4.	OTB arising from paranasal sinuses	8 [1], 1 [11]	9
5.	Tubercular dacryoadenitis	8 [1], 3 [10], 1 [12], 1 [13]	13
6.	Scrofuloderma orbit and adnexa	1 [15]	1
7.	Lupus vulgaris orbit and adnexa	2 [14], 1 [16]	3

## Conclusions

OTb, though rare, should form a part of the differential diagnosis of orbital lesions in a high TB endemic country like ours. Biopsy still remains the mainstay of diagnosis.

### Consent

Written informed consent was obtained from the patient/s for the publication of this report and any accompanying images.

## Abbreviations

MTb: *Mycobacterium tuberculosis*; OTb: orbital tuberculosis.

## Competing interests

The authors declare that they have no competing interests.

## Authors’ contributions

KB was involved in the conception, design, drafting of the manuscript and revisions. SSB, MM and CJT were involved in the acquisition of data and drafting of the manuscript. All the authors have read and approved the final manuscript.
